# The Influence of Millet Flour on Antioxidant, Anti-ACE, and Anti-Microbial Activities of Wheat Wafers

**DOI:** 10.3390/foods9020220

**Published:** 2020-02-19

**Authors:** Anna Jakubczyk, Paula Ćwiek, Kamila Rybczyńska-Tkaczyk, Urszula Gawlik-Dziki, Urszula Złotek

**Affiliations:** 1 Department of Biochemistry and Food Chemistry, University of Life Sciences, Skromna 8, 20-704 Lublin, Poland; anna.jakubczyk@up.lublin.pl (A.J.); paula.cwiek@onet.pl (P.Ć.); urszula.gawlik-dziki@up.lublin.pl (U.G.-D.); 2 Department of Environmental Microbiology, Laboratory of Mycology, The University of Life Sciences, Leszczyńskiego Street 7, 20-069 Lublin, Poland; kamila.rybczynska-tkaczyk@up.lublin.pl

**Keywords:** millet flour, wafers, antioxidant, rheological properties, biological compounds

## Abstract

The aim of the present study was to investigate antioxidant, angiotensin converting enzyme (ACE) inhibitory, and anti-microbial activities of wheat wafers enriched with 1%, 2%, or 3% (*w/w*) of millet flour (M1, M2, or M3, respectively). All samples were characterized by a richer composition of protein, polyphenols, flavonoids, phenolic acids, and reducing sugar in comparison with the control sample. The highest content of the components, i.e., 1.03 mg mL^−1^, 0.021 mg mL^−1^, 2.26 mg mL^−1^, 0.17 µg mL^−1^, and 0.63 mg mL^−1^, respectively, was detected in sample M3. The same sample was characterized by 803.91 and 42.79% of water and oil absorption capacity, respectively. The additive did not change the rheological features of the wafers. The 3% addition of millet flour to the wafer formulation induced the highest antioxidant activity against DPPH, Fe^2+^ chelation, and ACE inhibitory activity of hydrolysates (IC_50_ = 191.04, 0.46, and 157.73 µg mL^−1^, respectively). The highest activities were determined in the M3 fraction <3.0 kDa (IC_50_ = 3.46, 0.26, and 16.27 µg mL, respectively). In turn, the M2 fraction was characterized by the highest antimicrobial activity against *Listeria monocytogenes* with a minimum inhibitory concentration (MIC) value of 75 µg mL^−1^.

## 1. Introduction

Cereal products are the most common part of nutrition and are an important source of nutrients for the populations of both developed and developing countries. They are a good source of carbohydrates, protein, and fiber and contain vitamins, e.g., vitamin E, thiamin, riboflavin, niacin, vitamin B_6_, and folate, as well as minerals such as potassium, calcium, magnesium, or zinc [1]. Consumption of cereal products is associated with a lower risk of cardiovascular diseases, type 2 diabetes, obesity, and arteriosclerosis. The impact of this plant food on human health is determined by the cereal species and the processing methods.

Nowadays, problems with nutrition are involved in energy balance disorders. Consumers prefer ready-to-eat products, but this type of food is associated with high content of fat and calories. It is important that products with low calories and rich in bioactive compounds should be consumed in the daily diet. The latest epidemiological studies have indicated that consumption of excessive amounts of meat products is one of the main risk factors of cardiovascular diseases, obesity, cancers, and other dysfunctions of the organism [2,3,4].

One of the most common health problems worldwide is hypertension, which is a consequence of obesity and oxidative stress. Moreover, hypertension is associated with excessive activity of the rennin-angiotensin-aldosterone system (RAAS), which plays an important role in hormone regulation or maintenance of water and electrolyte balance. The main and best-recognized enzyme in RAAS is the angiotensin-I converting enzyme (ACE), which hydrolyzes inactive angiotensin I to active vasoconstrictor angiotensin II [5]. Therefore, reduction of ACE activity in an organism is important for regulation of blood pressure and water balance. Some synthetic inhibitors of ACE activity (e.g., lisinopril, enaparil, or ramipril) are commonly used as drugs for treatment of cardiovascular diseases and high blood pressure [6,7,8]. Another main factor in the development of hypertension is oxidative stress, i.e., imbalance between generation of reactive oxygen species (ROS) in the organism and antioxidant mechanisms. In the case of excessive activity of adipose tissue caused by its overgrowth, active particles such as pro-inflammatory cytokines, free fatty acids, angiotensinogen, and reactive oxygen species (ROS) are produced. All of them are involved in increased blood pressure and reduced vasodilation, fluid retention, and/or increased vascular stiffness. High content of ROS with reduced activity of antioxidant systems causes a number of diseases, including cancer, inflammation, diabetes, or cardiovascular disorders. Therefore, it is reasonable to find food compounds that can protect against the destructive effects of oxidative stress [9].

Wheat wafers are popular products among consumers used as a snack or part of meals. Wheat flour is deficient in some essential amino acids and other nutrients; therefore, to improve their nutritional value, wheat products can be enriched with protein, fiber, or bioactive compound-rich [10] or fiber-rich products [11,12]. Millet grains are a good source of dietary fiber and non-gluten proteins (kafirins) and have a high level of phytochemicals. Moreover, millet products are characterized by a low glycemic index; thus, they can especially be used by patients suffering from diabetes and carbohydrate metabolism disorders. Many studies have revealed that regular intake of millet products is associated with various health benefits such antiinflammatory, antioxidative, hypoglycemic, and cholesterol-lowering properties [13].

The constantly changing lifestyle and the occurrence of diseases of civilization have stimulated a demand for novel and enriched foods produced from various non-wheat flours. Therefore, the aim of this study was to determine the influence of addition of whole millet grain flour on the properties (antioxidant, ACE inhibitory, and anti-microbial) of wheat wafers.

## 2. Materials and Methods

### 2.1. Materials

The millet grains (*Panicum miliaceum* L.) were purchased from The Horticulture and Nursery Industry (PNOS) in Ożarów Mazowiecki, Poland. 

### 2.2. Preparation of Wafers

**Figure 1:**
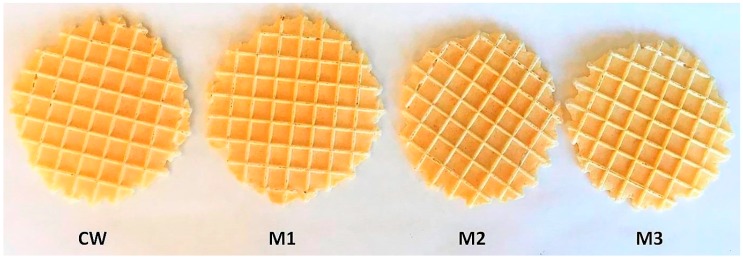
Wheat wafers (CW) with addition of millet grain flour (1%, 2%, 3%; M1, M2, M3, respectively).

Wafers were prepared by As-Babuni Company Sp. z o.o. (Niemce, Poland). Four types of wafers were prepared. The wheat flour was replaced with milled grain flour: average protein 11.0 g/100 g; ash 2.2 g/100 g; moisture content 11% (1%, 2%, 3%; M1, M2, M3, respectively). Wheat flour wafers without millet flour were used as a control (CW). The wafers were prepared by mixing the following ingredients: flour 50 kg (type 500: average protein 14.3 g/100 g; ash 0.5 g/100 g; moisture content 13.5%), water 65 L, oil 3 L, milk powder 2.15 kg, ammonium bicarbonate 0.4 kg, baking powder 0.4 kg, whey 1 kg, and lecithin 0.9 L to a homogenous structure and baking at 142 °C (ø 12.0 cm); (Figure 1).

### 2.3. Nutrient Compounds

#### 2.3.1. Preparation of Extracts 

About 10 mL of water were added to 0.5 g of the tested material and shaken at room temperature for 30 min. After that, the samples were centrifuged 8000× *g*, 10 min. The supernatant was decanted into a dry test tube; next, 10 mL of water were added to the residue, and the mixture was shaken for 30 min at room temperature. The samples were centrifuged for 10 min at 8000× *g*. The supernatants were combined and made up to a volume of 25 mL with distilled water. All extracts were made in triplicate.

#### 2.3.2. Protein Content in the Extracts

The protein content was determined according to the Bradford method [14].

#### 2.3.3. Peptide Content in the Extracts

The Adler-Nissen [15] method with L-leucine as a standard was used.

#### 2.3.4. Total Phenolic Content in the Extracts

The amount of total phenolic was determined using Folin–Ciocalteau reagent [16]. The amount of total phenolic content was calculated as gallic acid equivalent (GAE).

#### 2.3.5. Reducing Sugar Content in the Extracts

The reducing sugar content before hydrolysis was determined with the 3,5-Dinitrosalicylic acid (DNS) method [17].

### 2.4. Texture

The texture profile analyses of the wafer samples (62 × 77 mm) from the midsection of the wafers were performed using a texture analyzer (TA-XT Plus, Stable Micro Systems Ltd., Surrey, UK) with a 3 mm diameter cylindrical probe and a test speed of 1.0 mm s^−1^. The texture profile was determined using Texture Expert 1.05 software (Stable Microsystems). The other parameters were defined as: pre-test speed 1.0 mm s^−1^, post-test speed 1.0 mm s^−1^, and trigger force 5 g. The texture parameters recorded were hardness, fracturability, and puncture strength.

### 2.5. Colour

The color parameters of the wafers were measured using an Envisense NH310 colorimeter and the CIE Lab scale. The total color difference (ΔTC) of the wafers was calculated using formula (1), where L_0_*, a_0_*, and b_0_* are color parameters of control wafers:

$$\Delta \mathrm{TC}\;=\sqrt{{\left(L^{*}-L_{0}^{*}\right)}^{2}+{\left(a^{*}-a_{0}^{*}\right)}^{2}+{\left(b^{*}-b_{0}^{*}\right)}^{2}}$$

### 2.6. Functional Properties

#### 2.6.1. Water Absorption Capacity (WAC)

WAC of the prepared samples was determined according to the method described by Khattab and Arntfield [18] with modification as follows: each 0.5 g sample was added to 15 mL of distilled water and stirred for 5 min using a magnetic stirrer. The suspension was then centrifuged at 5000 × *g* for 30 min, and the supernatant was measured in a 10 mL graduated cylinder. WAC was determined as the difference between the initial volume of water added to the sample and the measured volume of the supernatant after centrifugation.

#### 2.6.2. Oil Absorption Capacity (OAC)

OAC was determined according to Khattab and Arntfield [18] with modification. One gram of the meal was mixed with 5 mL of oil in a centrifuge tube and allowed to stand at room temperature for 30 min. It was then centrifuged at 15000× *g* for 15 min. The volume of oil on the sediment was measured. OAC was calculated as milliliters of oil absorbed per gram of meal.

### 2.7. Nutrients Compounds

#### 2.7.1. Extract Preparation

About 10 mL of water was added to 0.5 g research material and shaken at room temperature during 30 min. After that, samples were centrifuged 8000× *g*, 10 min. The supernatant was decanted into a dry test tube, and to the residue was added 10 mL of water and shaken for 30 min in room temperature. The samples were centrifuged 10 min, 8000× *g*. The supernatants were combined and made up to a volume of 25 mL with distilled water. All extracts were made in triplicate.

#### 2.7.2. Protein Content of Extract

The protein content was determined according to Bradford method [14].

#### 2.7.3. Peptides Content of Extract

The method used was according to Adler-Nissen [15] method with L-leucine as a standard.

#### 2.7.4. Total Phenolic Content of Extract

The amount of total phenolic was determined using Folin–Ciocalteau reagent [16]. The amount of total phenolic content was calculated as gallic acid equivalent (GAE).

#### 2.7.5. The Reducing Sugar Content of Extract

Then, the reducing sugar content before hydrolysis was determined by using the DNS method [17].

### 2.8. In Vitro Hydrolysis

In vitro digestion of the wafers was carried out with the method described by Jakubczyk et al. [19]. The hydrolysates were clarified by centrifugation at 8000 g for 10 min (MPW, 350R, Poland) and kept at −20 °C.

All hydrolysates were centrifuged with Amicon Ultra-15 Centrifugal Filter Units, Merck Millipore (Membrane NMWL, 3 kDa), and fractions <3.0 kDa were obtained. 

### 2.9. Antioxidant Activity

#### 2.9.1. ABTS^•+^

Antiradical activity against 2,2’-Azino-bis(3-ethylbenzthiazoline-6-sulfonic acid (ABTS^•+^) was determined with the method described by Re et al. [20]. 

#### 2.9.2. DPPH^•^

Antiradical activity against di(phenyl)-(2,4,6-trinitrophenyl)iminoazanium (DPPH^•^) was determined using the method proposed by Brand-Williams, Cuvelier, and Berset [21]. 

#### 2.9.3. Fe^2+^ Chelating Activity

The method developed by Decker and Welch [22] was used to investigate the ferrous ion (II) chelating ability of the samples. 

### 2.10. ACE Inhibitory Activity Assay 

ACE inhibitory activity was measured with the spectrophotometric method using o-phtaldialdehyde as described by Jakubczyk et al. [19]. 

### 2.11. Antimicrobial Properties 

The hydrolysates and the peptide fraction of wafers were tested against the following bacteria: *Escherichia coli* ATCC 25922, *Staphylococcus aureus* ATCC 29737, *Listeria monocytogenes* ATCC BBA-2660, *Bacillus cereus* ATCC 14579, *Salmonella enteritidis* ATCC 4931, and yeast *Candida albicans* ATCC 90028. The strains were obtained from the American Type Culture Collection (ATCC, distributors: LGC Standards, Łomianki, Poland) and stored at 4 °C. All strains were cultured at 37 °C on Nutrient Broth (NB) medium. 

#### 2.11.1. Determination of the Minimum Inhibitory Concentration (MIC) 

Serial two-fold dilutions of wafer samples, hydrolysates, and peptide fraction were made with Mueller Hinton Broth (MHB) to yield final concentrations ranging from 40 to 2.5 mg mL^−1^, 2 to 0.125 mg mL^−1^, and 300 to 18.75 µg µL^−1^ respectively, and transferred into 96-well plates. A bacterial suspension (100 µl) prepared from an overnight culture was adjusted to inoculation of 10^8^ CFU mL^−1^. Then, 100 μL of the bacterial culture were added. The wells with MHB or yeast culture were the negative and the positive control, respectively. The plates were incubated at 37 °C for 48 h. The minimal inhibitory concentration (MIC) is an indication of the lowest concentration of the tested extracts that prevents visual growth of microorganisms. 

#### 2.11.2. Estimation of Biotoxicity Against *L. Monocytogenes* ATCC BBA-2660 Using Resazurin Reduction Assays

Resazurine reduction assays were performed to estimate biotoxicity against *L. monocytogenes* ATCC BBA-2660. Resazurin is a non-toxic water-soluble dye previously applied in bacterial viability studies [23]. This assay is based on detection of the metabolic activity of cells. The redox dye resazurin (7-hydroxy-3H-phenoxazin-3-one 10-oxide) enters the cell in the oxidized form (blue), where it is converted to a reduced form-resorufin (pink). The reduced and oxidized forms of resazurin can be measured separately with a spectrophotometer and used to determine the reduction capability of cells, which reflects the mitochondrial function and the cell viability and shows time- and concentration-dependent cell growth inhibition. After MIC estimation (2.10.1.), 20 μL of a 60 μM resazurin solution in phosphate buffered saline (PBS) buffer were added to each well. After incubation (2 h, 37 °C), the viability of cells was monitored by measuring absorbance at 570 nm (reduced) and 600 nm (oxidized) [23] and calculating bacterial viability (in percentages) against the control (bacterial growth without samples).

### 2.12. Statistical Analysis

All determinations were performed in triplicate. Statistical analysis was carried out using STATISTICA 13.1 for mean comparison using Tukey’s test at the significance level α = 0.05.

## 3. Results

Generally, the addition of the millet flour to the wafer formulation influenced the content of bioactive compounds (Table 1). The M3 sample was characterized by the highest content of all determined compounds, i.e., protein, polyphenols, flavonoids, phenolic acids, and reducing sugar (1.03 mg mL^−1^, 0.021 mg mL^−1^, 2.26 mg mL^−1^, 0.17 µg mL^−1^, and 0.63 mg mL^−1^, respectively). In all cases, the differences in the content of bioactive compounds were statistically significant, except the reducing sugar content, i.e., 0.62 mg mL^−1^ in M2 and 0.62 mg mL^−1^ in M3, which showed no statistically significant differences.

The millet flour-enriched wafers were characterized by higher water and oil absorption capacities than wafers without this additive (control sample). According to the data presented in Table 1, the addition of 1% millet flour had no statistical influence on WAC, whose value was 644.92% vs. 631.08% in the control. The highest WAC was determined for M3 (803.91%). However, only M3 was characterized by a statistically significant difference in OAC (42.79%) compared with the control sample.

According to the data shown in Table 2, the addition of the millet flour had no significant difference on the color parameter of the products. However, ΔTC of the color increased with the amount of the millet flour added to the wafers. The highest value was noted for M3 (4.33).

We also investigated hardness, fracturability, and puncture strength as the main rheological parameters of the wafers (Table 2). The wafers millet flour-enriched were characterized by lower hardness than the CW sample, and the lowest value was determined for M3 (856.68 g). In turn, the addition of the millet flour exerted no influence on the fracturability of the wafers. All samples had lower values than CW but these values were not statistically significant.

**Figure 2:**
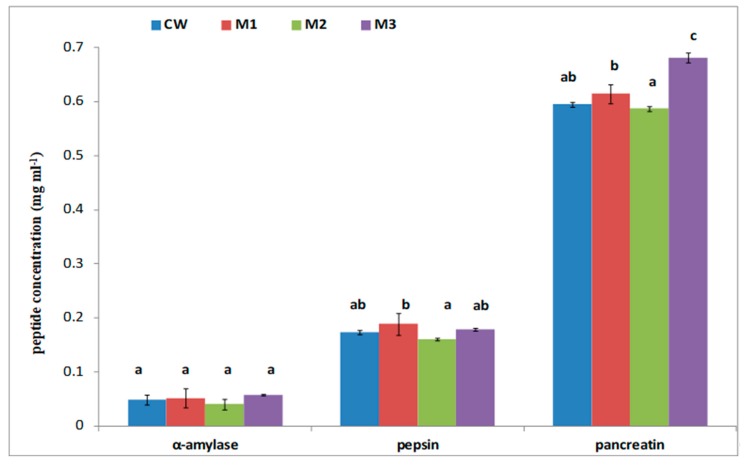
Peptide content in each step of hydrolysis. Different letters indicate significant differences.

The wafers were in vitro hydrolyzed in gastrointestinal conditions. Figure 2 shows changes in the peptide content after each step of the hydrolysis process. After α-amylase hydrolysis, the content of peptides was not statistically different and was below 0.1 mg mL^−1^ for all samples. Next, pepsin hydrolysis increased the peptide content in all hydrolysates. Nevertheless, no statistically significant changes were noted in any samples. After the last step of hydrolysis, the highest peptide content was determined for M3; it was 0.68 mg mL^−1^, which was significantly higher than in CW (0.59 mg mL^−1^).

After the hydrolysis process, antioxidant, ACE inhibitory, and antimicrobial properties of hydrolysates and fractions with <3.0 kDa molecular mass were determined. As shown in Table 3, the addition of the millet flour to the wafer formulation had an influence on all properties. All hydrolysates were characterized by lower antioxidant activity against ABTS^·+^ compared with CW, whereas higher antioxidant activity against DPPH was determined for hydrolysates obtained from the millet flour-enriched wafers. The lowest IC_50_ value for antioxidant activity against DPPH was noted for M3 = 191.04 µg mL^−1^. The lowest Fe^2+^ chelating activity was determined for M3 hydrolysates as well (0.46 mg mL^−1^), but this value did not show a statistically significant difference compared with the M2 hydrolysates (0.49 mg mL^−1^). Moreover, there was no relationship between the amount of the millet flour and the ACE inhibitory activity of the hydrolysates. The M1 hydrolysates were characterized by a higher IC_50_ value (205.76 µg mL^−1^) than that in the case of the CW hydrolysates (163.93 µg mL^−1^). In the case of M2 and M3, IC_50_ was lower than in CW (155.76 and 157.73 µg mL^−1^, respectively), but the difference between these values had no statistical significance.

Table 3 also shows antioxidant, ACE inhibitory, and antimicrobial properties of fractions with <3.0 kDa molecules. The fraction obtained from the CW hydrolysates was characterized by the highest antioxidant activity against ABTS^·+^ with the IC_50_ value of 26.67 µg mL^−1^. In turn, the fraction obtained from M3 exhibited the highest anti-DPPH^·^, Fe^2+^ chelating, and ACE inhibitory activities, with the IC_50_ values determined at 3.46 µg mL^−1^, 0.26 mg mL^−1^, and 16.27 µg mL^−1^, respectively.

**Figure 3:**
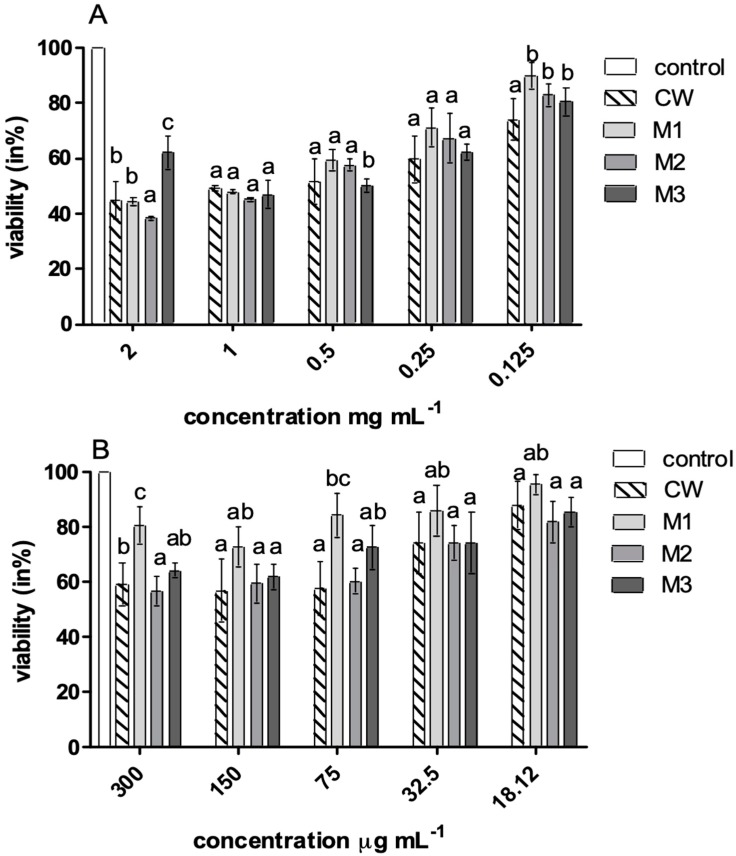
Viability (in %) of *L. monocytogenes* ATCC BBA-2660 against hydrolysates (**A**) and peptide fractions (**B**); control—growth of bacteria without hydrolysates or peptide fractions, CW—wheat wafers, M1, M2, and M3—wheat wafers enriched with 1%, 2%, or 3% (*w/w*) of millet flour, respectively. Different letters indicate significant differences.

Antibacterial properties of the hydrolysates and the peptide fractions of the wafers were tested against the following bacteria: *Escherichia coli* ATCC 25922, *Staphylococcus aureus* ATCC 29737, *Listeria monocytogenes* ATCC BBA-2660, *Bacillus cereus* ATCC 14579, *Salmonella enteritidis* ATCC 4931 and yeast *Candida albicans* ATCC 90028. The results showed that the hydrolysates and the peptide fractions had certain antimicrobial activity only against *L. monocytogenes* ATCC BBA-2660 (Table 3). The MIC values for the tested bacterial strain in the presence of hydrolysates and peptide fractions were in the range of 0.25–0.50 mg/mL and 75–150 µg/mL, respectively (Table 3). Among the tested hydrolysates and fractions, the CW and the M2 samples were the most effective inhibitors of *L. monocytogenes* ATCC BBA-2660 growth. This was also confirmed by the resazurin assay, which showed that the growth of *L. monocytogenes* ATCC 25922 treated with hydrolysates and peptide fractions was inhibited. In the case of the hydrolysates, the growth inhibition of the tested bacteria ranged from 10% to 68% and depended on the hydrolysate concentrations. Samples were classified as having an antimicrobial effect when they resulted in bacterial growth reduction of ≥40% in the resazurin reduction assay. The highest growth inhibition of *L. monocytogenes* ATCC 25922 was noted for the CW and the M2 samples (Figure 3A). In the case of the peptide fractions, the highest bacterial growth inhibition was noted for the CW and the M2 samples (Figure 3B).

## 4. Discussion

Millet grains as a source of bioactive compounds were investigated by our research team previously [24]. Since millet grain proteins are rich in bioactive peptides that may be involved in inhibition of development of diseases, the aim of this study was to determine their influence on antioxidant and anti-ACE activity as well as the antimicrobial properties of wafers enriched with millet flour. The results indicated that the addition of the millet flour had a significant effect on the content of bioactive compounds. The wafers with the millet flour were characterized by higher content of the tested compounds, except reducing sugars (Table 1). This may suggest that these products are a good source of bioactive compounds with health benefits especially useful for prevention of oxidative stress-related diseases [25]. Bakery products are the most common snacks due to their low cost, good taste, variety, and texture. In addition, their composition can be modified to obtain functional food recommended to patients suffering from cardiovascular diseases, celiac disease, or obesity [26]. The most important components that may increase health-promoting properties are polyphenols [27], anthocyanins [28,29], or peptides [30]. In addition to changing the properties and the taste, they can alter the rheological properties of final products. The enhanced ability of bakery products to absorb water and oil may help to improve the structure, improve mouth feel, enhance flavor retention, improve binding of additives such as whole grains or pieces of fruit, and reduce moisture and fat losses of food products [31]. The level of WAC of food protein is affected by size, shape, and composition of proteins, especially the hydrophilic–hydrophobic balance of amino acids in the molecules as well as the presence of lipids and hydrophilic carbohydrates [32]. In our study, the millet flour-enriched wafers were characterized by higher water and oil absorption capacities compared to wafers without the millet flour, and the values increased with the amount of the added flour. It should be noted that the AOC parameters were not increased significantly, which may indicate a larger share of hydrophilic amino acids in proteins.

Enrichment of food products with various types of additives may also affect the consumer quality of the finished product. Color is one of the product features influencing consumer choice and acceptability. In this study, the addition of the millet flour to the wafer formulation had no influence on their color (Table 2). This means that CW had the same L* (lightness), a* (redness), and b* (yellowness) values as M1, M2, and M3 samples. Moreover, the total color change increased with the increasing level of millet flour addition. These results correspond well with those reported by Baumgartner et al. [33], where the total color change increased with an increasing level of untreated or dephytinized oat brans. This parameter was also increased in cookies obtained with refined wheat flour and raw/roasted flaxseed flour blends [34].

The millet flour-enriched wafers were characterized by lower hardness and fracturability, although no statistically significant differences were determined in the latter parameter. These results correspond well with those reported by Ghaboos et al. [35], where texture profile analysis of the hardness of sponge cakes showed that the product became harder with the levels of added pumpkin powder increasing from 0 to 20%. This feature may be associated with the protein content in the final product. The interaction between protein and starch as the main ingredients in the wafers decreased the number of amylopectin chains during the process of baking, which in turn inhibited water vapor release and increased hardness [36]. This process may also have exerted the main effect on the puncture strength of the wafers.

Food proteins provide organisms with not only amino acids but also bioactive peptides that may have a beneficial effect on the function of the organism [37]. Peptides are released from proteins during hydrolysis in the gastrointestinal tract. In our study, we determined the content of peptides also after α-amylase hydrolysis. It should be noted that our previous study demonstrated that the peptide content increased after this step of hydrolysis, which indicated some sugar linkages in the proteins [38]. The highest peptide content was noted after the pancreatic hydrolysis of M3. The 3% addition of the millet flour improved the potential bioaccessibility of peptides released from proteins. Polyphenols are another important group of bioactive food compounds. There are many studies of food enriched with polyphenol-rich ingredients [10]. Polyphenols are nonpolymeric phytochemical compounds containing phenolic hydroxyl groups in their structures and exhibiting various properties such as antioxidant, anti-inflammatory, and anticancer activities or inhibiting the development of metabolic diseases, e.g., diabetes, obesity, or hypertension [39,40]. In our study, the highest total phenolic content was noted for M3; it corresponded well with the antioxidant and the anti-ACE activity (Table 3). A similar increase in the antioxidant activity and the total phenolic content compared to the control were observed in flaxseed flour-supplemented cookies [41]. Free radicals, e.g., superoxide anion (·O2^−^), hydroxyl radical (^·^OH), and hydrogen peroxide (H_2_O_2_), are natural metabolic products. Under homeostasis, reactive oxygen radicals released in cell-safe amounts play a role as mediators and regulators of many cellular processes [42]. Production of excessive amounts of free radicals by cells and weakening of the natural antioxidant systems are correlated directly with molecular markers of many disease conditions, including diabetes, atherosclerosis, neurodegenerative diseases, inflammation, and cancer [43]. Oxidative stress with other inflammatory factors may initiate changes in cardiovascular function and structure such as endothelial dysfunction, cardiac dysfunction, cardiac fibrosis, and vascular remodeling [44]. The present results indicate that all samples (hydrolysates and fractions with <3.0 kDa molecular mass) contained some bioactive compounds that were electron donors and could react with free radicals to convert them to more stable products and terminate the radical chain reaction. There was no correlation between the hydrolysates and the three kDa fractions and the DPPH assay. This may be due to the difference between the compositions of the hydrolysates and the fractions. As reported by other authors, food ingredients have an influence on protein digestibility and hydrolysate compounds. Moreover, we expected that the anti-DPPH activity would be increased proportionally in the bioactive compounds in the samples. It should be noted that the quantity and the activity of fractions depend on the food matrix [45,46,47]. Recent studies on the antioxidant activity of food are complemented with the analysis of ACE inhibitory activity, since oxidative stress and hypertension have become the most serious health problems affecting society [48].

Consumption of antioxidants from food sources is potentially effective in promotion of human health, as antioxidation is known to mitigate the adverse effects of free radicals. However, there have also been numerous reports suggesting that the effects of dietary antioxidants depend on many factors such as body condition, dose, duration of administration, source of bioactive compounds, and other dietary factors [43,49]. Compared with traditional drugs commonly used for treatment of cardiovascular disease and inflammation, exogenous molecular antioxidants, especially peptides, are usually reported to exhibit reduced negative side effects and low toxicity [50].

Previous studies indicated antibacterial activity of plant protein hydrolysates and fractions [51,52]. However, the antimicrobial activity of protein hydrolysates or peptides extracted from rich sources of protein is less well known, particularly the activity against pathogenic bacteria causing harmful food-borne human diseases. In turn, there have been increasing and uncontrolled outbreaks or incidents of food-borne diseases associated with pathogens. Our results are in agreement with a previous study describing inhibition of *L. monocytogenes* growth upon treatment with protein hydrolysates and peptide fractions [53]. It should be emphasized that foods supplemented with protein hydrolysates or peptide fractions are not only functional foods with health benefits but also a source of substances that inhibit the growth of pathogenic bacteria responsible for food poisoning.

## 5. Conclusions

Bakery snacks are very often chosen by consumers because of their taste, price, and variety. Their composition can be modified to increase their health benefits and technological properties. In the present study, different hydrolysates and fractions with <3.0 kDa were extracted from millet flour-enriched wheat wafers. Since oxidative stress and hypertension are the most common health problems for populations of developing countries, the study was focused on determination of antioxidant and anti-ACE activities in the product. This research confirmed the antimicrobial activity of the protein hydrolysates and fractions against pathogenic bacteria causing harmful food-borne human diseases. The overall data suggest that the wafers enriched with millet flour can be used as a potential source of natural antioxidant, antimicrobial, and anti-ACE peptides in formulation of functional foods.

## Figures and Tables

**Table 1:** Characteristic of the content of bioactive compounds and functional properties of wafers.

	Color				Rheological Properties		
Sample	L*	a*	b*	ΔTC	Hardness (g)	Fracturability (mm)	Puncture Strength (N/mm^2^)
CW	61.92 ± 5.36 ^a^	8.77 ± 1.04 ^a^	23.75 ± 1.95 ^a^	-	911.02 ± 108.53 ^a^	68.35 ± 0.39 ^a^	2.63 ± 0.32 ^a^
M1	64.80 ± 2.92 ^a^	8.53 ± 1.02 ^a^	23.93 ± 1.96 ^a^	2.89 ± 0.14 ^a^	870.26 ± 140.58 ^a^	67.60 ± 0.18 ^b^	2.16 ± 0.42 ^a^
M2	64.84 ± 1.87 ^a^	8.76 ± 1.27 ^a^	25.04 ± 1.46 ^a^	3.19 ± 0.08 ^b^	862.85 ± 119.84 ^a^	67.49 ± 0.63 ^b^	2.47 ± 0.60 ^a^
M3	65.96 ± 1.83 ^a^	7.30 ± 1.55 ^a^	23.22 ± 2.51 ^a^	4.33 ± 0.06 ^c^	856.68 ± 139.26 ^a^	67.06 ± 0.42 ^b^	3.7 ± 0.90 ^a^

All values are mean ± standard deviation for triplicate experiments. Different letters indicate significant differences (α = 0.05).

**Table 2:** Influence of millet flour on color characteristics and rheological properties of wafers.

Sample	Bioactive Compound Content					Functional Properties	
	Protein (mg mL^−1^)	Polyphenols (mg mL^−1^)	Flavonoids (mg mL^−1^)	Phenolic Acids (µg mL^−1^)	Reducing Sugars (mg mL^−1^)	WAC (%)	OAC (%)
CW	0.69 ± 0.017 ^a^	0.015 ± 0.002 ^a^	0.85 ± 0.07 ^a^	0.13 ± 0.004 ^a^	0.59 ± 0.004 ^a^	631.08 ± 14.54 ^a^	38.43 ± 1.44 ^a^
M1	0.75 ± 0.008 ^b^	0.019 ± 0.003 ^b^	1.88 ± 0.012 ^b^	0.14 ± 0.002 ^b^	0.61 ± 0.003 ^b^	644.92 ± 18.36 ^a^	39.12 ± 1.48 ^a b^
M2	0.99 ± 0.008 ^c^	0.020 ± 0.002 ^c^	1.96 ± 0.013 ^c^	0.16 ± 0.003 ^c^	0.62 ± 0.002 ^b c^	714.77 ± 20.38 ^b^	41.34 ± 1.24 ^a b^
M3	1.03 ± 0.012 ^d^	0.021 ± 0.003 ^d^	2.26 ± 0.010 ^d^	0.17 ± 0.004 ^d^	0.63 ± 0.008 ^c^	803.91 ± 28.71 ^c^	42.79 ± 1.86 ^b^

All values are mean ± standard deviation for triplicate experiments; Different letters indicate significant differences (α=0.05). WAC, water absorption capacity; OAC, oil absorption capacity.

**Table 3:** Antioxidant, angiotensin converting enzyme (ACE) inhibitory, and antibacterial properties of hydrolysates and fractions with <3.0 kDa molecules.

Sample	Properties (IC_50_ ug mL^−1^)				Antibacterial Activity (*L. monocytogenes* ATCC BBA-2660)
	ABTS^·+^	DPPH^·^	Fe^2+^ Chelation	ACE	MIC
	Hyrolysates				
CW	67.22 ± 1.78 ^a^	404.26 ± 3.18 ^a^	0.58 ± 0.03 ^a^	163.93 ± 1.25 ^a^	0.25 *
M 1	80.08 ± 1.99 ^b^	287.23 ± 2.41 ^b^	0.62 ± 0.01 ^a^	205.76 ± 1.57 ^b^	0.50 *
M 2	101.54 ± 2.15 ^c^	198.09 ± 1.18 ^c^	0.49 ± 0.01 ^b^	155.76 ± 2.08 ^c^	0.50 *
M 3	113.34 ± 1.06 ^d^	191.04 ± 2.83 ^d^	0.46 ± 0.02 ^b^	157.73 ± 1.69 ^c^	0.50 *
	Fractions <3.0 kDa				
CW	26.67 ± 0.73 ^a^	31.71 ± 0.98 ^a^	0.32 ± 0.01 ^ab^	21.11 ± 0.77 ^a^	75 **
M 1	62.58 ± 1.13 ^b^	129.45 ± 1.01 ^b^	0.33 ± 0.02 ^ab^	24.17 ± 0.51 ^b^	-
M 2	39.42 ± 1.08 ^c^	227.01 ± 1.58 ^c^	0.31 ± 0.02 ^a^	15.67 ± 1.03 ^c^	75 **
M 3	41.50 ± 2.014 ^d^	3.46 ± 0.14 ^d^	0.26 ± 0.01 ^c^	16.27 ± 0.89 ^c^	150 **

MIC, minimum inhibitory concentration; * mg mL^−1^, ** µg mL^−1^; All values are mean ± standard deviation for triplicate experiments. Different letters indicate significant differences (α = 0.05).
